# Activity of MMV Pandemic Response Box compounds against *Mycobacterium ulcerans* viability and mycolactone production

**DOI:** 10.1128/spectrum.03968-25

**Published:** 2026-08-19

**Authors:** Louisa Warryn, Daniel H. Paris

**Affiliations:** 1 Swiss Tropical and Public Health Institute 30247, Allschwil, Switzerland; 2 University of Basel 27209, Basel, Switzerland; University of Manitoba, Winnipeg, Manitoba, Canada

**Keywords:** *Mycobacterium ulcerans*, mycolactone inhibition, GW611920X, pradigastat, alexidine, fusidic acid, MMV Pandemic Response Box

## Abstract

*Mycobacterium ulcerans* causes Buruli ulcer (BU), a skin neglected tropical disease, largely through the expression of a potent cytotoxin known as mycolactone. As mycolactone is central for BU pathogenesis, it follows that targeting its production with drugs could be one means of controlling the infection. However, this has not yet been attempted due to the difficulty of applying typical mycolactone-detecting assays, such as mass spectrometry, to routine drug screening to identify suitable drugs for this purpose. In this repurposing study, we screened the Pandemic Response Box, an open-source collection of 400 compounds compiled by the Medicines for Malaria Venture and the Drugs for Neglected Diseases Initiative, using a highly sensitive and specific mycolactone capture ELISA that we have previously developed. By combining this mycolactone-detecting assay with the routinely used resazurin microplate assay, we identified hits (such as pradigastat) that are potential starting points for further development of antivirulence drugs for BU. We also showed the activity of alexidine and fusidic acid against *M. ulcerans* viability, the first report of this activity to our knowledge. In addition, we reliably reidentified drugs known to be active against *M. ulcerans* (such as bedaquiline, Q203, clofazimine, sutezolid, and epetraborole), thus validating our screening approach.

**IMPORTANCE:**

Mycolactone is a key requirement in the pathogenesis of Buruli ulcer (BU). Without this unique toxin, *Mycobacterium ulcerans* only causes a transient infection, as the immune system is able to clear the bacteria when mycolactone is absent. There has, therefore, been a long-standing interest in targeting mycolactone to develop new drugs against BU, but a lack of simple drug screening tools has been a major obstacle to this goal. Here, we used our screening platform, which comprises a simple mycolactone-detecting ELISA combined with a standard resazurin microplate assay, to identify a set of hits from the Medicines for Malaria Venture Pandemic Response Box that could reduce mycolactone expression even without affecting *M. ulcerans* viability. This screening methodology will contribute to further development of antivirulence drugs for BU.

## INTRODUCTION

Of the 21 neglected tropical diseases (NTDs) on the World Health Organization (WHO) list, *Mycobacterium ulcerans* disease—or Buruli ulcer (BU)—is one of the most neglected, and it continues to cause significant morbidity in over 30 tropical and subtropical countries. BU is the third most prevalent mycobacterial disease after tuberculosis and leprosy, and majority of the cases occur in parts of Africa, Australia, and the Western Pacific islands. With climate change and increasing anthropogenic land changes (1, 2), there is an increased risk of the disease expanding its range and reemerging in historically quiescent regions (3). The mainstays of effective BU control are the diagnosis and treatment of every case. The WHO-recommended treatment is an 8-week regimen of once-daily rifampicin and twice-daily clarithromycin. As the routes of transmission are still unknown in most of the highly endemic regions, and as no effective vaccination exists, the options to prevent disease remain highly limited. Thus, methods to improve BU diagnosis and treatment are actively researched, particularly to develop treatment regimens that are shorter, easier to administer, and with fewer side effects.

BU is a skin NTD characterized by chronic lesions in skin and soft tissue, causing widespread destruction in the absence of quick and effective treatment. These destructive lesions are caused by the effects of the potent polyketide exotoxin, known as mycolactone, that *M. ulcerans* secretes into its surroundings (4). *M. ulcerans* diverged from a common ancestor with the closely related *Mycobacterium marinum* following acquisition of an approximately 174-kb plasmid that encodes the machinery for the synthesis of its unique mycolactone toxin (5). All symptoms of BU are attributable to direct or secondary effects of mycolactone, including the tissue necrosis, local hyposensitivity, and localized immunosuppression that characterize the disease (4). As mycolactone is central to BU pathogenesis, and experimental infection of mycolactone-deficient mutants results in a self-limiting granulomatous infection reminiscent of *M. marinum* infection (5), it follows, therefore, that an approach aiming at incapacitating mycolactone could be a means of treating the infection. It is already known that depletion of mycolactone levels in tissues—as happens during standard antibiotic treatment when the bacterial load is reduced—is necessary for healing to commence (6, 7). However, the difficulty in quantifying mycolactone in samples using the typical chromatographical methods and mass spectrometry has thus far made it impossible to screen for compounds that may inhibit mycolactone production without killing the bacteria.

Given the paucity of funding for BU drug development, drug repurposing is the most cost-effective means of developing new BU drugs, and such repurposing efforts are facilitated by the availability of open-source compound collections, such as those put together by the Medicines for Malaria Venture (MMV, Geneva, Switzerland). The Pandemic Response Box (PRBox) is one such collection, comprising 400 antibacterial, antifungal, and antiviral compounds collated by MMV and the Drugs for Neglected Diseases Initiative (Geneva, Switzerland) (8). In this study, we assessed the potential of the PRBox collection as a source of active compounds for new BU drug development. We screened the 400 PRBox compounds for activity against different *M. ulcerans* strains using a resazurin microplate assay as previously reported (9) and largely reidentified drugs and compounds already known to adversely affect *M. ulcerans* viability. Additionally, in a first assessment of its kind, we screened these compounds for their effect on mycolactone production, using the highly specific and sensitive mycolactone capture immunoassay that we previously developed (10). This pioneering effort demonstrated the feasibility of developing antimycolactone drugs in a cost-effective and medium-throughput manner, and identified some hits with potential to be starting points for further development of such antivirulence drugs.

## MATERIALS AND METHODS

### Bacteria and media

Three low-passage *M. ulcerans* clinical isolates were used for the various analyses. The Cameroonian strain S1013 is an established standard in our laboratory (11), which, together with the Australian isolate S1252, represented the classical *M. ulcerans* lineage in this study, while the Japanese strain S1324 represented the ancestral lineage. All three bacterial strains are low-passage clinical isolates (<3 passages) with no discernible impairment in mycolactone production. Bacteria were cultivated at 30°C in Middlebrook 7H9 medium, supplemented with 1% glycerol, 0.1% casein, and 10% oleic acid, albumin, dextrose, catalase (OADC).

Middlebrook 7H9 medium supplemented with 0.2% glycerol and 10% OADC was used for the compound screening assays.

### Compounds

All 400 compounds in the PRBox were independently dissolved in 10 μL of sterile cell-culture-grade DMSO upon receipt from the MMV to give 10 mM stock solutions. The compounds were stored at −20°C until needed.

### Screening protocol

#### Resazurin assays

**Fig 1:**
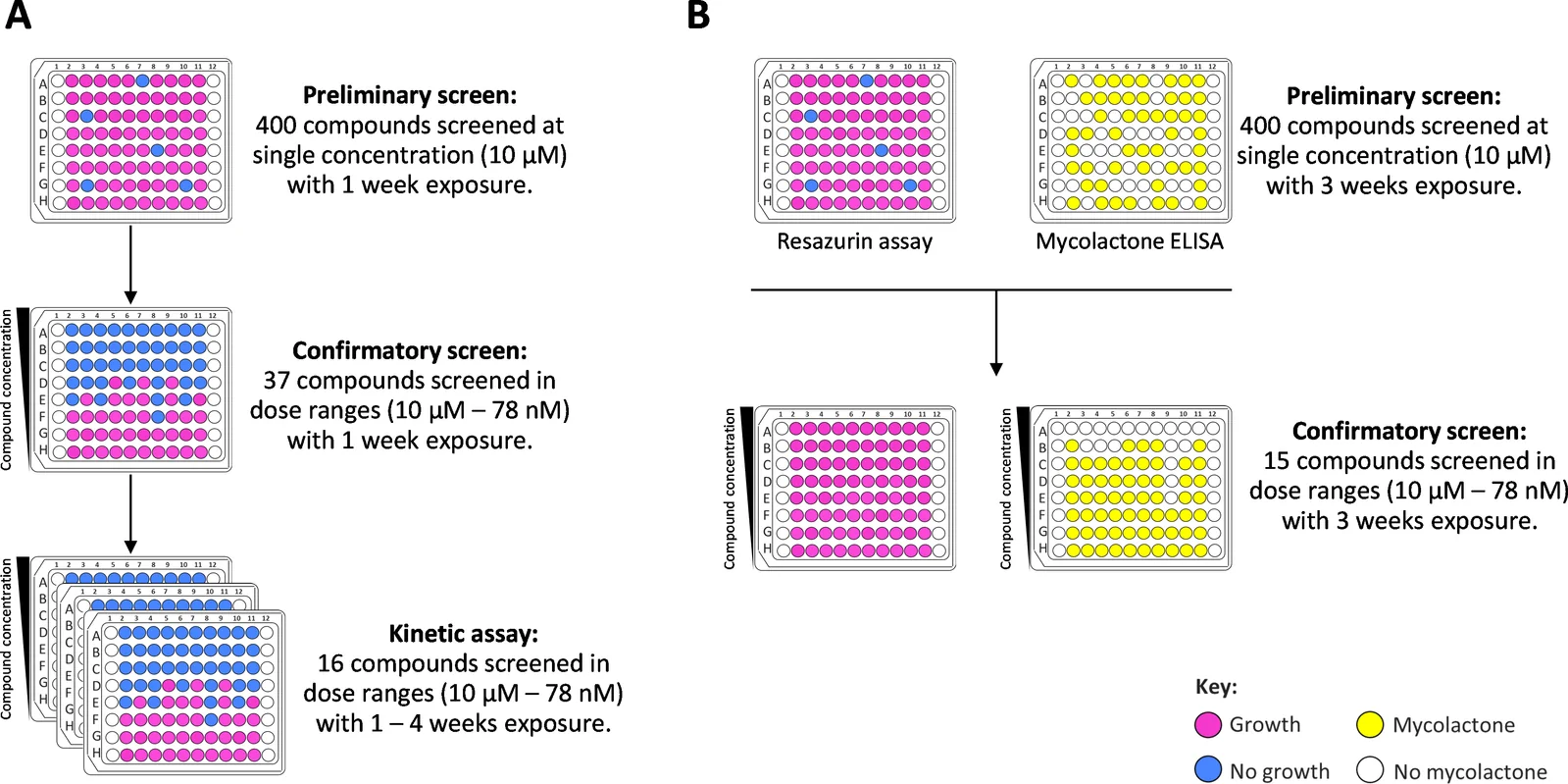
Screening approach. (**A**) Resazurin microtiter assay was used to assess the effects of the 400 MMV PRBox compounds on the viability of *M. ulcerans*. Compounds that showed ≥50% reduction in viability in the preliminary screen were analyzed in dose ranges in confirmatory screens. Highly active compounds were further analyzed in a resazurin-based kinetic assay. (**B**) Resazurin microplate assay and mycolactone capture ELISA were used to assess the effects of the 400 MMV PRBox compounds on mycolactone expression. Compounds that reduced mycolactone expression by ≥50% relative to untreated bacteria but with no effect on *M. ulcerans* viability were further analyzed in dose ranges in confirmatory screens.

The 400 compounds were initially screened for activity against *M. ulcerans* strain S1013 at a single concentration of 10 μM (in duplicates) using a resazurin microtiter assay, as previously described (9) (Fig. 1A). Briefly, *M. ulcerans* at an initial optical density at 600 nm (OD_600 nm_) of 0.04 was exposed to test compounds for 1 week at 30°C. Thereafter, 10% vol/vol of a 0.125 mg/mL resazurin solution was added, and the cultures were incubated for an additional 3 days, after which the fluorescence was measured (Ex_540 nm_/Em_588 nm_). The metabolic activity was determined relative to the included *M. ulcerans* drug-free control. This assay was repeated for all compounds which showed up to 50% reduction in metabolic activity, to confirm anti-*M*. *ulcerans* activity. Highly active compounds were then screened in dose ranges against the three *M. ulcerans* strains to check for lineage-specific activity.

All highly active compounds were further analyzed in a resazurin-based kinetic assay that we have previously shown to give comparable results to CFU enumeration (12). Here, cultures representing both *M. ulcerans* lineages (initial OD_600 nm_ of 0.04) were incubated at 30°C with a dose range of test compounds for 1, 2, or 4 weeks. At each time point, 10% vol/vol of a 0.125 mg/mL resazurin solution was added, and then cultures were incubated for an additional 3 days, after which the fluorescence was measured (Ex_540 nm_/Em_588 nm_). The metabolic activity was determined relative to the included *M. ulcerans* drug-free control. The minimum inhibitory concentration was defined as the concentration that led to 50% reduction in metabolic activity (MIC_50_) and was calculated from the dose-response curves using GraphPad Prism (version 8.2.1; GraphPad Software, San Diego, CA).

#### Mycolactone assessment

All 400 compounds were also screened (in duplicates) to assess their effects on *M. ulcerans* mycolactone production (Fig. 1B). Here, *M. ulcerans* strain S1013 (initial OD_600 nm_ of 0.04) was washed twice with the medium to remove accumulated mycolactone in the culture, and then exposed to 10 μM of each test compound for 3 weeks at 30°C. Thereafter, the culture was split in halves, and one-half was used for the resazurin assay as described above. To the other half, absolute ethanol was added to give an ethanol concentration of 33%, and plates were incubated at ambient temperature for 3 days. This allowed for extraction of mycolactone from the *M. ulcerans* extracellular matrix, and the 3-day extraction duration ensured that both the resazurin and mycolactone readouts could be done simultaneously to avoid experimenter bias (all compounds were also only referred to by code names during all screening assays). Afterwards, mycolactone detection was done via a mycolactone-specific capture ELISA as previously described (10). Briefly, MaxiSorp ELISA plates (Thermo Scientific, catalog number 442404) were coated with 2 μg/mL of a mycolactone capturing monoclonal antibody (mAb) and blocked with blocking buffer (PBS with 0.05% Tween-20 [PBST] with 5% skimmed milk). After washing the plates three times with washing buffer (PBST), 50 μL triethanolamine buffer (0.2 M TEA, pH 7.5) was added to each well of the ELISA plates, and then the entire contents of the extraction plates were transferred to the ELISA plates. This reduced the effective ethanol concentration to 25%, a concentration predetermined to be compatible with the ELISA. After incubation and washing steps, biotinylated mycolactone detecting mAb (3 μg/mL) was added and incubated. Bound biotinylated mAb was detected using HRP-conjugated streptavidin (SouthernBiotech; catalog number 7105-05, 1:4,000 dilution). Signal was developed with TMB (KPL SeraCare, catalog number 5120-0047), after which the reaction was stopped with 0.5 M sulfuric acid. Absorbance was measured at 450 nm with an ELISA microplate reader (Tecan Sunrise), and the results were illustrated using GraphPad Prism (version 8.2.1, GraphPad Software).

For all compounds, the results of the resazurin assay and the mycolactone ELISA were compared. All compounds that reduced the amount of detected mycolactone by ≥50% relative to the included drug-free controls but which did not have any detrimental effect on metabolic activity were retested in dose ranges to determine the ED_50_, defined as the minimum concentration that reduced detected mycolactone by 50% relative to drug-free controls.

## RESULTS AND DISCUSSION

### Activity against *M. ulcerans* viability

#### Initial screening

**Fig 2:**
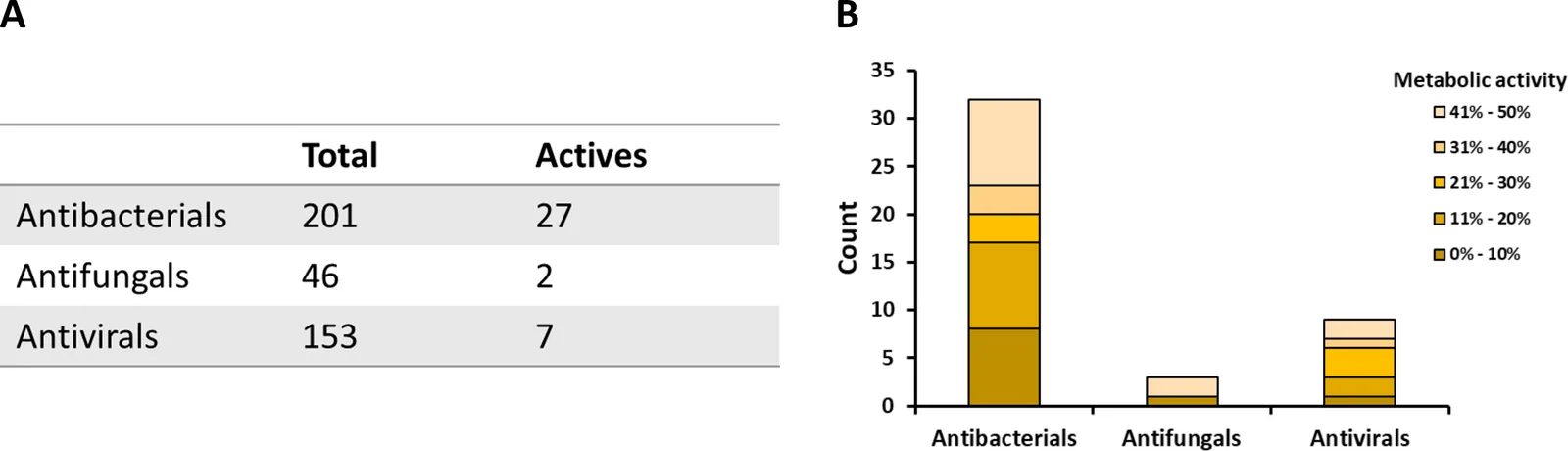
(**A**) Number of compounds with activity against *M. ulcerans* viability as determined by the resazurin assay. (**B**) Categories of active compounds.

Of the 400 compounds in the PRBox, 36 (9%) reduced the metabolic activity of *M. ulcerans* S1013 by ≥50%. Of these, 27 were antibacterials, some of which are known or have been previously reported to be active against *M. ulcerans*, e.g., bedaquiline (MMV689758), Q203 (MMV687696), and clofazimine (MMV687800) (Fig. 2). Two antifungals also had activity against this strain in this initial screen—alexidine (MMV396785) and OSU-03012 (MMV1578560), the most active of which was alexidine. The most active antiviral from this initial screen was 1,1-dioxide 1-thioflavone (MMV1006203).

#### Activity against classical vs ancestral lineage strains

**Fig 3:**
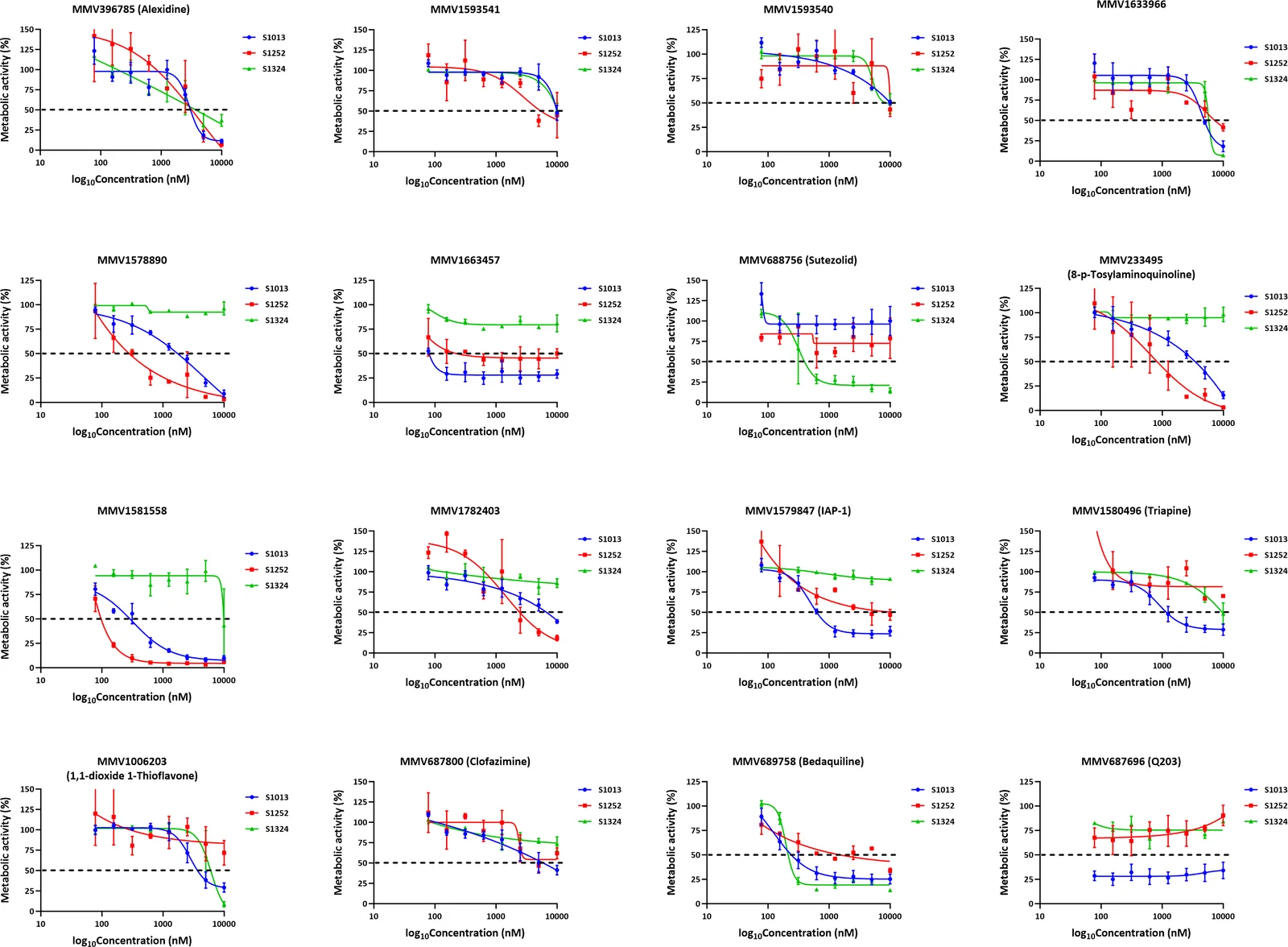
Activity of PRBox compounds against three *M. ulcerans* strains representing the classical (S1013 and S1252) and ancestral (S1324) lineages. Activity was assessed via the resazurin microplate assay following 1 week of exposure to test compounds. The means of three independent experiments are shown, with the error bars representing the SEM. The MIC_50_ is indicated by the dotted line in each graph.

Following these initial screens, dose ranges of these 36 compounds (which included bedaquiline, Q203, and clofazimine as positive comparators) as well as sutezolid were tested against three *M. ulcerans* strains representing both lineages. Sutezolid (MMV688756) was included in this test as, although reported to be active against *M. ulcerans* (13), it was consistently inactive against the classical lineage strain S1013 in the preliminary screens. Rifampicin was also tested as an additional positive control. Following these screens, 21 of the 37 compounds were excluded, as they were found to only have activity at the highest concentration tested (10 μM) against the three strains. Some of the compounds had higher activity against the classical lineage strains than against the ancestral lineage strain. Interestingly, the reverse was the case with sutezolid (MMV688756), which showed much higher activity against the ancestral lineage strain (Fig. 3). Rifampicin, which was also tested as an additional positive control, gave MIC_50_ values of 0.15–0.97 µM for the three strains (0.34 µM for S1013, 0.15 µM for S1252, and 0.97 µM for S1324).

**Fig 4:**
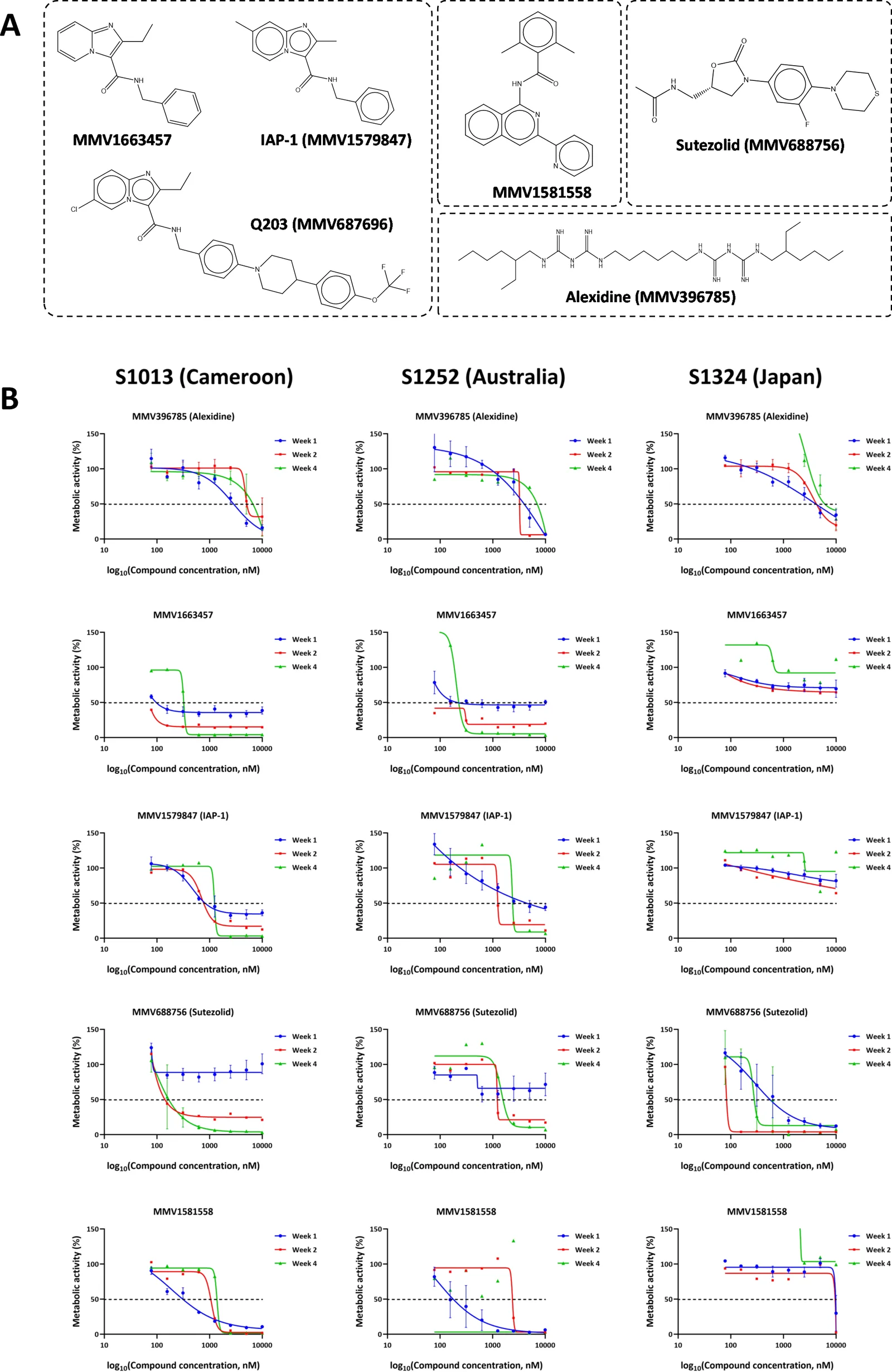
(**A**) MMV PRBox compounds with the highest activity against *M. ulcerans*. (**B**) Activity against three *M. ulcerans* strains representing the classical and ancestral lineages was assessed via the resazurin microplate assay following 1–4 weeks of exposure to test compounds. The MIC_50_ is represented by the dotted line.

For the time-kill assay, 16 compounds (including the three known compounds, bedaquiline, Q203, and clofazimine) were tested against the three *M. ulcerans* strains for up to 4 weeks. There were five compounds with the best activity against *M. ulcerans* viability, as shown in Fig. 4; the remaining compounds only had MIC_50_ values between 5 and 10 μM, with several only being active within the first week of exposure. Like Q203, both MMV1663457 and IAP-1 (MMV1579847) are imidazopyridine carboxamide (IPA) compounds with structural similarity to Q203 (Fig. 4). This may explain their higher activity against classical lineage *M. ulcerans* strains compared to the ancestral lineage strain. Q203 has been shown to target QcrB, the *b* subunit of the mycobacterial respiratory cytochrome *bc*_1_:*aa*_3_ (cyt-*bc*_1_:*aa*_3_) supercomplex, which is the primary cytochrome oxidase with which mycobacteria carry out oxidative phosphorylation. Similar to *Mycobacterium tuberculosis*, ancestral lineage *M. ulcerans* strains have a secondary cytochrome oxidase that can compensate for blockade of the primary cytochrome oxidase by Q203 and related compounds, while this secondary cytochrome oxidase is non-functional in the classical lineage *M. ulcerans* strains (14). As this drug class has been extensively studied, we did not do any further tests on MMV1663457 and IAP-1 (MMV1579847).

The 2,6-dimethyl-N-(3-pyridin-2-ylisoquinolin-1-yl)benzamide compound MMV1581558 (Fig. 4) showed good activity against the classical lineage *M. ulcerans* strains (MIC_50_ values of 84–192 nM) but had only modest activity against the ancestral lineage strain (MIC_50_ ≈ 10 μM). This compound has been previously shown to be quite active against *Schistosoma mansoni in vitro* but with only modest *in vivo* activity (15), had modest activity against *Plasmodium falciparum* (16), and was part of identified compounds active against *Toxoplasma gondii* (17) in previous tests of the MMV PRBox. As these are eukaryotic parasites, activity against the prokaryotic *M. ulcerans* may not necessarily be due to similarity of molecular targets, although more research is required to evaluate the potential of this compound as a novel scaffold for further development. It could well be that all four diseases could benefit from further development of MMV1581558, and given the epidemiological overlap of these tropical diseases, it may be instrumental in integrated disease control efforts.

Sutezolid (MMV688756) has been shown to have activity against *M. ulcerans* in the mouse model of BU (13). It was interesting to note that, in our assays, it had no effect on the viability of the classical lineage strains following the standard 1-week exposure, but already had activity against the ancestral lineage within the same time frame. Activity against the classical lineage strains only became apparent upon longer exposure. As this drug has already been studied in the *in vivo* BU mouse footpad model, we did not do any further tests on sutezolid.

Alexidine (MMV396785) is a biguanide disinfectant and has a broad spectrum of activity, with reports of it being active against several pathogen types, including bacteria, fungi, helminths, and other eukaryotic parasites (18, 19). Not surprisingly, therefore, it also had activity against all three *M. ulcerans* strains in our assays (MIC_50_ values of 2.7–4.7 μM). Although too toxic for oral (systemic) use, it may be a useful adjunct to BU treatment as a topical agent.

Thus, after going through rounds of activity testing, as is the norm in standard drug discovery and development for *M. ulcerans*, we reidentified previously reported compounds such as bedaquiline, Q203, and other IPAs, clofazimine, and sutezolid, thus validating our testing protocol. The fact that these are all known antimycobacterial compounds supports the approach of repurposing drugs from the tuberculosis development pipeline to devise new *M. ulcerans* treatment paradigms. The only novel compounds identified in this compound collection from these rounds of testing were MMV1581558 and alexidine, both of which could be potential starting points for further development.

### Effect on mycolactone production

**Fig 5:**
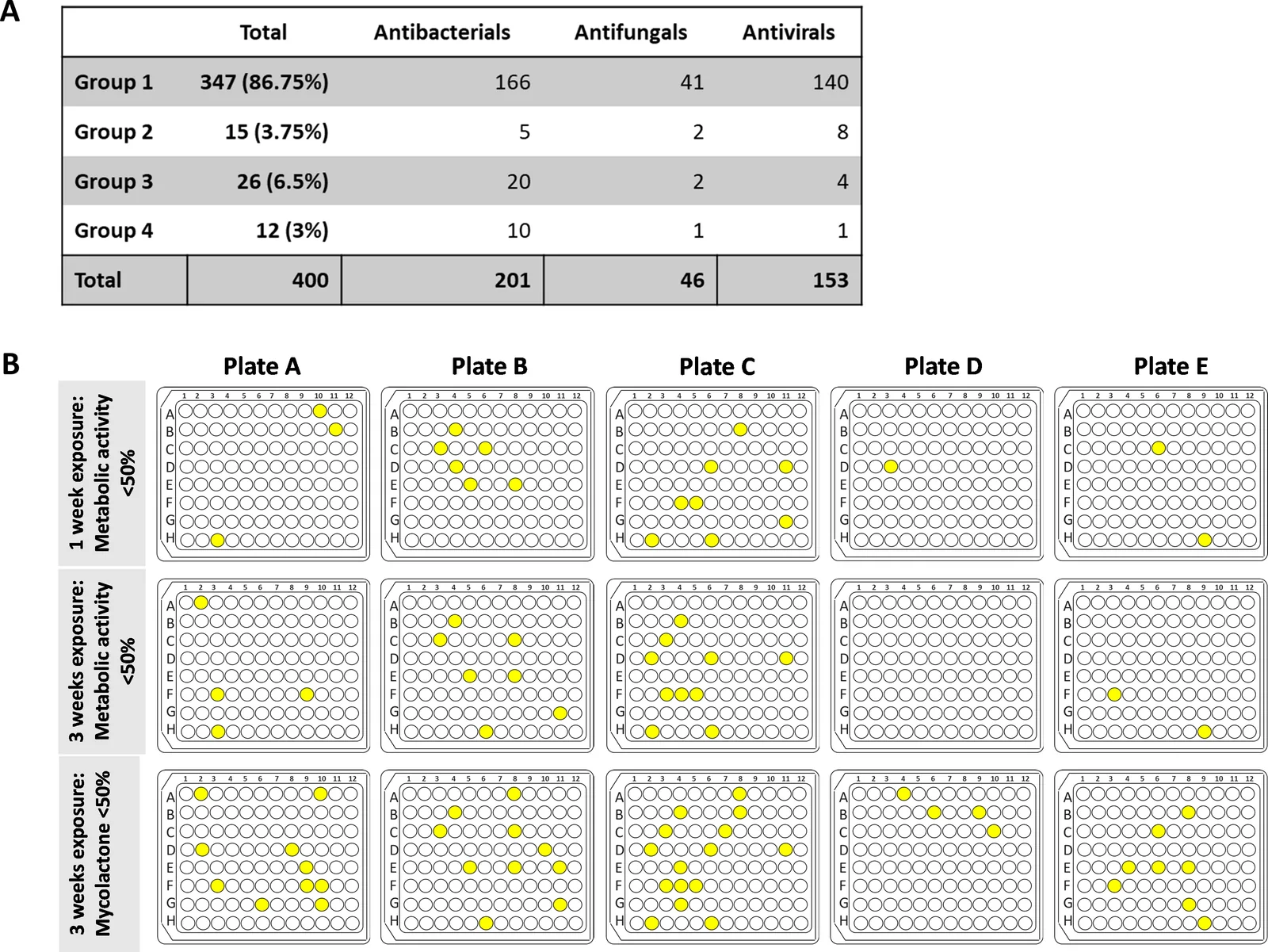
(**A**) Categories of MMV PRBox compounds based on effect on *M. ulcerans* viability and mycolactone production. (**B**) MMV PRBox compounds affecting mycolactone detection (group 2 compounds). The results of both resazurin preliminary screens (i.e., 1- and 3-week exposure assays) and the mycolactone capture ELISA preliminary screen are shown. Test compounds were in 80 wells per plate (rows A–H, columns 2–11). Yellow circles signify wells containing active compounds.

In a next step, we tested the effects of the PRBox compounds on mycolactone production. For this series of analyses, all 400 compounds in the PRBox were tested to assess their effects on *M. ulcerans* viability following the standard 1-week testing paradigm (as described above) or a 3-week exposure; and in parallel, the relative amounts of detected mycolactone following the 3-week exposure were measured. All three readouts were used to determine the effect of the test compound on mycolactone production (Fig. 5 and Table S1). Consequently, each test compound could fall into one of four groups:

- Group 1 compounds are those that are completely inactive against *M. ulcerans* because they reduced neither *M. ulcerans* viability nor mycolactone amounts. The majority of the compounds (347, 86.75%) were in this group.

- Group 2 compounds are those that only reduced the amounts of detectable mycolactone but which had little to no effect on *M. ulcerans* viability at any of the time points. In other words, the reduction in mycolactone was not due to the killing of the bacteria. These are the compounds that potentially specifically reduce mycolactone and are the main targets of this screening protocol. Only a minority of compounds (15, 3.75%) fell into this group.
- Groups 3 and 4 compounds are those with activity against *M. ulcerans* viability at one (group 3) or both (group 4) time points. Here, any reduction in mycolactone seen is likely due to bacterial inhibition. Few compounds (38, 9.5%) fell into these groups.
  - Group 3 compounds (26, 6.5%) reduced *M. ulcerans* metabolic activity following either a 1- or 3-week exposure. For 14 compounds in this group, there was only activity at the earlier time point, with *M. ulcerans* S1013 becoming less sensitive to these compounds with extended exposure. In most cases, mycolactone levels also increased as metabolic activity increased with time. The exceptions were MMV1633967, MMV000043 (tafenoquine), and MMV1578885 (PBTZ-169), where mycolactone levels remained suppressed even though the bacteria were no longer inhibited by the compounds. The remaining 12 compounds in this group only reduced *M. ulcerans* metabolic activity following prolonged exposure, with mycolactone reducing concomitantly. Among these were compounds already known to have activity against mycobacteria, including *M. ulcerans*, such as MMV688756 (sutezolid), as well as the fluoroquinolones MMV637659 (nadifloxacin) and MMV1579850 (sitafloxacin), and the boronic compound MMV1578566 (epetraborole) (20). The only compounds in this group previously not reported to have activity against *M. ulcerans* were MMV1633674 (retapamulin), MMV1580796 (rubitecan), MMV1578575 (fusidic acid), and MMV1580173 (trimetrexate). Both fusidic acid and trimetrexate had MIC_50_ values of approximately 2 μM but suppressed detected mycolactone by >90% (Table S1).
  - Group 4 compounds (12, 3%) showed sustained activity against *M. ulcerans*, with a concomitant reduction in detected mycolactone. They included the known antimycobacterial compounds bedaquiline, clofazimine, and Q203, as well as the active compounds described above (IAP-1, MMV1581558, etc.). The only outlier in this group was alexidine (MMV396785), which surprisingly showed no reduction in mycolactone levels but rather elevated levels relative to the untreated cells. The reason for this is unclear but could be related to the antibiofilm properties of this disinfectant (21); it is conceivable that alexidine may destabilize the abundant extracellular matrix of *M. ulcerans*, releasing the mycolactone contained therein, therefore allowing for more of the toxin to be detected by the assay. Further studies would be required if alexidine is to be further developed into a topical agent for BU treatment.

Of the 15 group 2 compounds (Table 1), pibenzimol (MMV020752) and fludarabine (MMV637413) showed the strongest suppression of detectable mycolactone (>90% suppression for both). However, as pibenzimol (also known as Hoechst dye 33258) and fludarabine are both reported to be cytotoxic (22, 23), further development as an oral BU treatment is rather unlikely. With >70% suppression, the next best mycolactone-suppressing compounds were the synthetic vitamin A derivative fenretinide (MMV001793) and GW611920X (MMV1578577). To ascertain that the effect on mycolactone levels was unrelated to cell density (i.e., that these were indeed group 2 compounds), we tested dose ranges of these compounds, incorporating measurement of OD_600 nm_ following exposure for three weeks, before splitting the culture in halves for the resazurin and mycolactone assays. We included the known antimycobacterial drugs rifampicin, clarithromycin, and streptomycin in these assays as positive controls and obtained MIC_50_ values of 0.38, 0.79, and 0.89 µM, respectively, which are in line with reported *in vitro* MICs (24).

**TABLE 1:** Compounds suppressing mycolactone expression (group 2 compounds)

Compound ID	Trivial name	% inhibition at 10 μM (relative to untreated cells)		
		Metabolic activity		Detected mycolactone
		1-week exposure	3-week exposure	3-week exposure
MMV020752	Pibenzimol	−4.5	26.6	93.6
MMV637413	Fludarabine	9.1	−5.2	92.2
MMV001793	Fenretinide	1.1	−2.1	79.4
MMV1578577	GW611920X^*a*^	−260.3	−2.9	73.5
MMV018362	Cinchophen	5.4	16.0	67.8
MMV1580504		8.6	6.1	66.0
MMV1578574	Eravacycline^*a*^	3.6	7.3	61.7
MMV1580498	Pradigastat	27.4	−3.5	61.6
MMV1578568	Gepotidacin	−29.9	2.9	60.2
MMV218827		11.6	2.3	57.8
MMV003137	Erythromycin	−36.7	13.6	57.1
MMV1634386		21.6	12.0	54.4
MMV394033		9.1	9.1	51.1
MMV1782354	Olorofim	−24.8	−4.8	50.6
MMV1580486	Pritelivir	−2.5	−0.7	49.7

^*a*^ These compounds were recategorized as group 3 compounds based on OD measurements.

**Fig 6:**
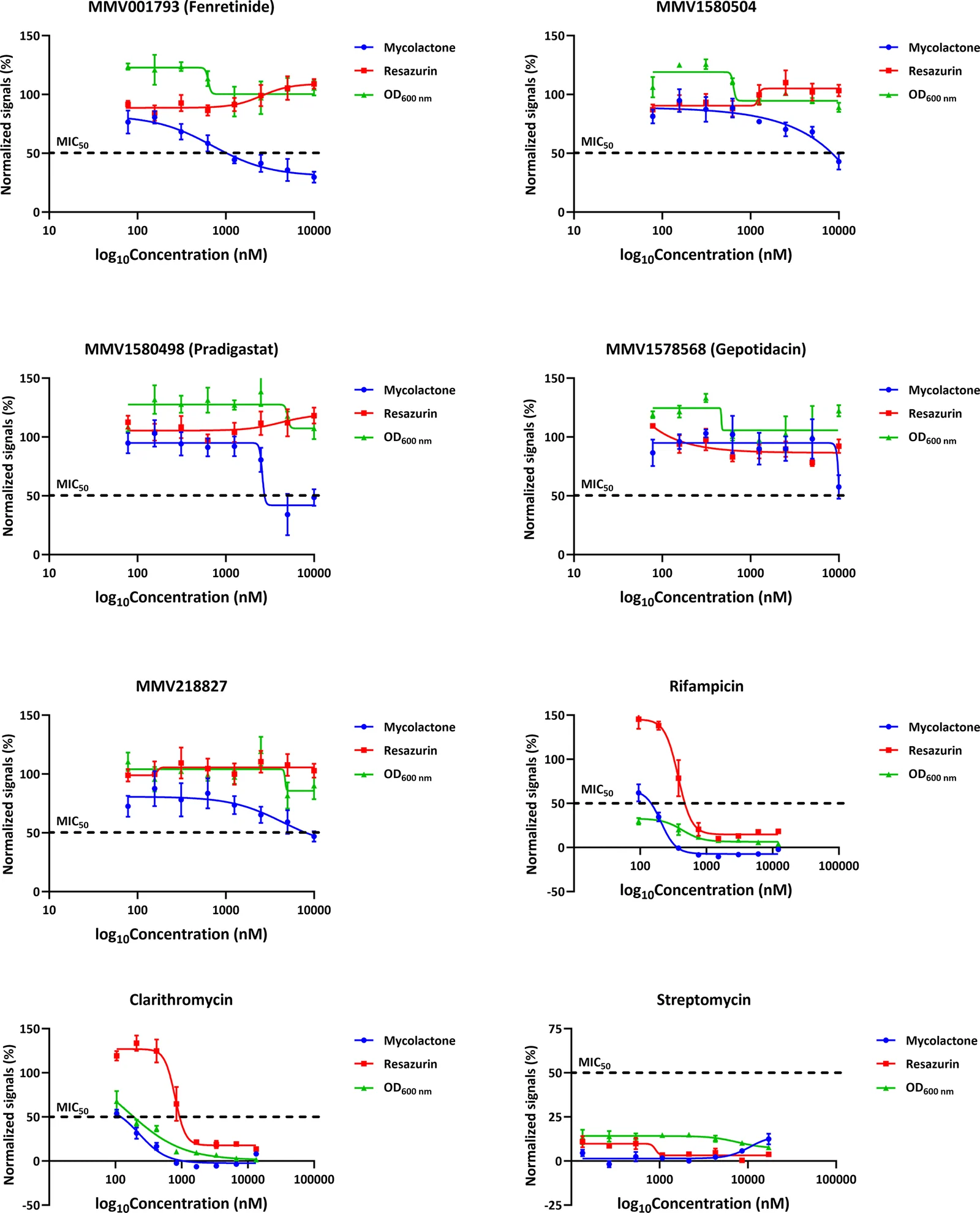
PRBox compounds suppressing mycolactone expression. Dose ranges of test compounds were assessed for their effects on *M. ulcerans* viability (as determined by resazurin reduction and OD_600 nm_) and mycolactone detection (as determined by capture ELISA [10]). Graphs show the means of triplicate experiments with error bars representing the SEM.

Only fenretinide and pradigastat showed a dose-dependent reduction of detectable mycolactone without affecting OD_600 nm_ or metabolic activity, with ED_50_ of 0.8 and 5.3 µM, respectively (Fig. 6); all the others only suppressed mycolactone at the highest concentration tested (i.e., ED_50_ ≈ 10 μM). Fenretinide has been investigated over the years as a potential treatment for various cancers and recently as a possible antiviral agent as it showed activity against dengue virus (25) and the helicase activity of the SARS-CoV-2 non-structural protein 13 (Nsp13) (26). It has been shown to preferentially accumulate in fatty tissue, and this affinity for fats may play a role in its suppression of the lipid-like mycolactone molecule. The known mechanisms of action of fenretinide include induction of apoptosis of cancer cells via ceramide- and ROS-mediated pathways, although further studies would need to be done to elucidate the mechanism(s) of its antimycolactone activity. Pradigastat is used to treat aberrant triglyceride metabolism and consequently has some antiobesity and antidiabetes effects. It selectively inhibits diacylglyceryl acyltransferase 1 (DGAT-1), which catalyzes the final step in triglyceride synthesis in human enterocytes and hepatocytes (27). It was also reported to have *in vitro* efficacy against hepatitis C virus replication, although this activity was not seen in a subsequent clinical study (28). Given its known target, it is predominantly being investigated as a host-directed treatment for viral infections; therefore, it is unclear what mechanisms may underlie the reduction in detectable mycolactone we observed. However, as we saw no evidence of mycolactone being outcompeted by either fenretinide or pradigastat in our ELISA (results not shown), the actual mechanism by which these compounds reduce the amount of detectable mycolactone is probably unrelated to an association with and sequestration of the toxin and is possibly related to the bacteria themselves. Given mycolactone’s lipid-like nature, it is probably noteworthy that both pradigastat and fenretinide interact with lipid pathways in mammalian cells, although as we have not done any formal mechanism of action studies at this time, it is premature to speculate on how this may impact *M. ulcerans* mycolactone.

In terms of repurposing for BU treatment, pradigastat is the more promising candidate, as fenretinide is reported to have low bioavailability, frequently caused visual side effects in clinical trials (29), and consequently has not been approved for clinical use for any of the indications for which it has been investigated. Thus, the potential for repurposing it into a new anti-BU treatment is quite low, since extensive and potentially costly derivatization and medicinal chemistry to overcome its shortcomings would be needed for such repurposing.

Both eravacycline (MMV1578574) and GW611920X (MMV1578577) showed a dose-dependent reduction in both OD_600 nm_ and mycolactone levels but had no effects on metabolic activity, indicating that the suppression of mycolactone was related to a reduction in bacterial biomass (Fig. S1). This led to both these compounds being recategorized as group 3 compounds. GW611920X may nevertheless be a promising candidate for further development, as it was previously shown to have low *in vitro* cytotoxicity (17) and to have activity against *M. tuberculosis* and a panel of non-tuberculous mycobacteria (NTMs), including *Mycobacterium abscessus* and *Mycobacterium chelonae* (30). Indole-2-carboxamides such as GW611920X are known to target mycobacterial cell wall formation by inhibiting trehalose monomycolate translocation during mycolic acid biosynthesis.

### Compound classes

Given the advantages of repurposing already licensed drugs compared to drugs still early in the development stages for NTDs such as BU, in this section, we are highlighting the activity of PRBox compounds belonging to established drug classes against *M. ulcerans*, both in terms of bacterial killing and inhibition of detectable mycolactone.

#### Antimycobacterials

Several compounds with known activity against mycobacteria were included in the PRBox. Of the older antimycobacterial drugs, isoniazid (MMV002169), pyrazinamide (MMV003249), and ethambutol (MMV687801) were all inactive against *M. ulcerans* at all time points tested, consistent with previous reports (31). The isoniazid derivative TSC-INH (MMV1782227) was also inactive. Clofazimine, currently used in treating leprosy, was quite active against *M. ulcerans* (MIC), as has been previously reported (24). Epetraborole (MMV1578898), which has activity against NTMs, was recently shown to also be active against *M. ulcerans* (20). For all these drugs, their activity or inactivity was confirmed both by their effects on *M. ulcerans* growth and on the detectable levels of mycolactone (Table 2).

**TABLE 2:** Activity of known representatives of known drug classes against *M. ulcerans*

Compound ID	Trivial name	% Inhibition at 10 μM (relative to untreated cells)		
		Metabolic activity		Detected mycolactone
		1-week exposure	3-week exposure	3-week exposure
Antimycobacterials				
MMV1782227	TSC-INH	7.3	−0.3	14.0
MMV687273	SQ109	28.0	−11.6	−23.3
MMV687800	Clofazimine	60.5	83.5	90.6
MMV688755	Pretomanid	1.1	7.1	−4.7
MMV1578898	Epetraborole	10.9	90.1	95.5
MMV688756	Sutezolid	1.5	92.2	92.5
MMV687801	Ethambutol	−76.9	−3.5	−2.3
MMV002169	Isoniazid	22.0	3.1	15.3
MMV687696	Q203	66.9	78.5	90.4
MMV003249	Pyrazinamide	16.6	1.8	41.2
MMV689758	Bedaquiline	75.2	85.1	79.3
Macrolides				
MMV003137	Erythromycin	−36.7	13.6	57.1
β-Lactams and β-lactamase inhibitors				
MMV002740	Cloxacillin	−51.7	−2.7	−17.6
MMV1578559	Cefoxitin	−158.9	−5.4	1.7
MMV002560	Cefditoren pivoxil	−150.9	13.6	6.6
MMV1613560	Avibactam	−26.5	0.7	6.4
MMV637945	Sulbactam	−32.4	−17.8	10.4
MMV1582382	Panipenem	19.8	1.8	−96.9
Aminoglycosides				
MMV002459	Tobramycin	6.8	52.4	86.6
Fluoroquinolones				
MMV637659	Nadifloxacin	−6.4	56.8	80.7
MMV1579850	Sitafloxacin	−18.8	56.7	85.0
MMV002350	Lomefloxacin	−34.4	7.5	9.9
MMV687798	Levofloxacin	−30.4	16.7	−28.0
Tetracyclines				
MMV1578574	Eravacycline	3.6	7.3	61.7
Azoles				
MMV1634492	Eberconazole	7.9	61.8	59.5
MMV002337	Fluconazole	22.4	10.8	29.0
MMV637533	Ketoconazole	7.1	35.4	11.3
MMV637528	Itraconazole	−28.9	8.7	20.5
MMV1634362	Ravuconazole	10.1	9.5	19.5
MMV1782224	Luliconazole	−14.6	−2.6	−54.8
MMV002722	Miconazole	29.9	43.9	−112.2
MMV003143	Fenbendazole	−70.6	−15.4	24.7
MMV002565	Oxfendazole	11.1	13.8	12.0
MMV001438	Lansoprazole	38.1	−1.3	26.9
MMV002015	Clemizole	38.6	9.2	−2.8
MMV1613562	Morinidazole	24.4	2.2	1.9
MMV002287	Secnidazole	32.5	5.3	25.4
Others				
MMV1578575	Fusidic acid	1.2	90.5	95.1
MMV000028	Trimethoprim	9.0	−5.3	7.5
MMV000051	Clindamycin	44.7	46.7	−50.8
MMV002354	Phosphomycin	26.3	18.5	28.5
MMV001726	Fusaric acid	50.8	14.0	27.5

#### Macrolides

The macrolide antibiotic clarithromycin is currently used in combination with rifampicin to treat BU; therefore, this drug class is already known to be active against *M. ulcerans*. Clarithromycin exhibits a bacteriostatic effect on *M. ulcerans*, with a reported MIC value of approximately 0.12 μg/mL (24). Erythromycin (MMV003137) was the only macrolide antibiotic included in the PRBox. However, it showed no effect on *M. ulcerans* viability at any time point tested. Interestingly, though, it did show modest antimycolactone effects, reducing detected levels of the toxin by >50% relative to untreated cells (Table 2).

#### β-Lactams and β-lactamase inhibitors

The penicillin compound cloxacillin (MMV002740), the cephalosporins cefoxitin (MMV1578559) and cefditoren pivoxil (MMV002560), the carbapenem panipenem (MMV1582382), as well as two β-lactamase inhibitors—avibactam (MMV1613560) and sulbactam (MMV637945)—were included in the PRBox. They were all inactive at all testing time points and did not reduce the amount of detected mycolactone (Table 2). β-Lactams are not known to be active on their own against mycobacteria but have been shown to be synergistic with other antimycobacterials, including the recent reports of synergistic activity of amoxicillin plus clavulanate with the current rifampicin-clarithromycin regimen against *M. ulcerans* (32).

#### Aminoglycosides

The aminoglycoside drug streptomycin, which is strongly bactericidal to *M. ulcerans*, was the previously used companion drug to rifampicin in BU chemotherapy. This drug class is, therefore, already known to have activity against *M. ulcerans*. Tobramycin (MMV002459), the only aminoglycoside included in the PRBox, had slight activity against *M. ulcerans* upon prolonged exposure but had much greater effects on mycolactone expression, reducing the level of detectable mycolactone by >80% (Table 2).

#### Fluoroquinolones

Four fluoroquinolones were included in the PRBox. Both nadifloxacin (MMV637659) and sitafloxacin (MMV1579850) were active against *M. ulcerans* strain S1013 and concomitantly reduced the level of detected mycolactone but only upon longer incubation (Table 2). Sitafloxacin was previously reported to be active against *M. ulcerans* both *in vitro* (MIC 0.125–0.5 μg/mL or 0.31–1.22 μM) (33) and *in vivo* (34), but there have not been any reports on the activity of nadifloxacin on *M. ulcerans*. Curiously, both levofloxacin (MMV687798) and lomefloxacin (MMV002350) were inactive against *M. ulcerans* at all time points tested in this study. They also had no dampening effect on the level of detected mycolactone. This is in contrast with *in vitro* data showing the activity of different fluoroquinolones (ofloxacin, ciprofloxacin, and sparfloxacin) against *M. ulcerans* (35) and clinical efficacy of rifampicin-fluoroquinolone combination therapy for BU (36), with levofloxacin being a part of standard BU chemotherapy in some BU-endemic areas (37). These disparate results could be due to differences in strains and testing methods used (some may need longer exposure than the 3 weeks used in the present study), and it is possible that some fluoroquinolones may be active only when used in combination with other drug classes.

#### Tetracyclines

Tetracycline antibiotics are not known to be active against *M. ulcerans*, and mycobacteria are generally resistant to first- and second-generation tetracyclines. Eravacycline (MMV1578574), the only included tetracycline antibiotic in the PRBox, is a third-generation tetracycline, and third-generation tetracyclines such as eravacycline, tigecycline, and omadacycline have been shown to have some activity against NTMs, such as *M. abscessus* (38, 39). Similarly, eravacycline had a slight effect on *M. ulcerans* viability at the later time point tested, with over 60% reduction in detectable mycolactone (Table 2 and Fig. S1). Interestingly, its effect on *M. ulcerans* viability was only apparent when OD_600 nm_ was measured alongside resazurin reduction.

#### Azoles

Antifungal azoles are drugs that target fungal cell wall biosynthesis, typically by inhibiting ergosterol biosynthesis. Chemically, these antifungals are imidazoles or imidazole derivatives, such as benzimidazoles or nitroimidazoles. Azoles in general were found to be largely inactive against *M. ulcerans*, with only croconazole having been active in a previous report (9). Regarding the present study, several azole compounds were included in the PRBox. Of the seven imidazoles in this collection, eberconazole (MMV1634492) was the only one with activity against *M. ulcerans* strain S1013 (Table 2). It was also previously shown to have transmission-blocking activities against *P. falciparum* in previous tests of the PRBox (16). Eberconazole is available as a 1% cream, making it a potential topical treatment for BU. There were four benzimidazole and two nitroimidazole compounds included in the PRBox. None of the two nitroimidazoles—morinidazole (MMV1613562) and secnidazole (MMV002287)—nor the four benzimidazoles had any appreciable activity against *M. ulcerans* in any of the screening assays. Lansoprazole (MMV001438) is a substituted benzimidazole used predominantly as a treatment for peptic ulcer. It is a prodrug that inhibits H^+^/K^+^-ATPase when exposed to acidic conditions, which explains its activity in treating peptic ulcers. However, when it undergoes sulfoxide reduction in the cytoplasm of *M. tuberculosis*-infected macrophages, it selectively inhibits the mycobacterial QcrB (40). Therefore, although it is expected to be active against *M. ulcerans*, the lack of activity seen in our screening exercises could be due to the fact that we did not use an intracellular screening paradigm, and therefore the necessary sulfoxide reduction (which activates its antimycobacterial activity) was not achieved.

#### Other drug classes

The steroid antibiotic fusidic acid (MMV1578575), fusaric acid (MMV001726), the lincosamide clindamycin (MMV000051), trimethoprim (MMV000028), and the phosphonic acid antibiotic phosphomycin (MMV002354) were all included in the PRBox. Both clindamycin and fusaric acid had weak (and in the case of the latter, transient) activity against *M. ulcerans* viability, and trimethoprim and phosphomycin were both inactive (Table 2). Conversely, fusidic acid showed good activity against *M. ulcerans* viability (MIC_50_ ≈2 μM) with >90% mycolactone suppression at the highest test concentration (ED_50_ ≈6.2 μM). Fusidic acid has been reported to be active against *M. tuberculosis* (in combination with other antibiotics) (41) and *Mycobacterium leprae* (42), but this is the first report of its activity against *M. ulcerans* to our knowledge. Fusidic acid could therefore be a potential addition to BU treatment, either orally administered or used topically, as there are topical preparations of this antibiotic.

### Conclusion

In this screening exercise, we assessed the effects of the 400 compounds in the MMV PRBox on *M. ulcerans* viability and on the levels of mycolactone as detectable by capture ELISA. For the first time, we have shown the feasibility of screening for compounds that could inhibit mycolactone production in a cost-effective way with reasonable throughput. Using our mycolactone detection assay and the standard resazurin drug screening protocol, we could reidentify known antimycobacterial drugs. Antimycobacterials known to be inactive against *M. ulcerans* were also inactive in both our assays, further validating our screening platform. This exercise further corroborates the approach of drug repurposing as a cost-effective means of developing new drugs for BU treatment. In particular, repurposing antituberculosis drugs is still a valid means of new BU drug development. Here, we reidentified known antimycobacterial drugs, such as clofazimine, bedaquiline, Q203 (telacebec), and sutezolid, which are all known to be active against *M. ulcerans*, as well as epetraborole, a previously identified drug active against NTMs that was recently shown to also be active against *M. ulcerans*. In this vein, open-source compound collections such as the PRBox are a valuable resource for such drug repurposing efforts.

One key output of this work was the identification of mycolactone-inhibiting compounds. Although most of the identified compounds were either too cytotoxic (e.g., pibenzimol, fludarabine, and fenretinide) or not effective enough (e.g., pradigastat) for further BU drug development, they may, however, be promising scaffolds for further development of safe and effective antivirulence drugs for BU treatment. This screening exercise also indicated that previously overlooked antibiotic classes, with respect to mycobacterial drug development, may be potential sources of novel antimycolactone drugs. This is a possibility that we intend to explore in a follow-up screening, targeting multiple representatives of known drug classes to identify drugs that could be repositioned as novel and safe antimycolactone drugs.

A potential limitation of our screening approach is that we do not use a co-culture methodology and, so, might miss some potentially active compounds. We saw a manifestation of this in this study, as we could not confirm the activity of lansoprazole against *M. ulcerans* since this prodrug needs intracellular activation. We also observed that incorporating OD measurements into the screening pipeline alongside resazurin reduction was beneficial in separating reduction in detectable mycolactone levels from bacterial biomass reduction. Nevertheless, our screening platform is robust and was able to correctly identify all known antimycobacterials that were included in the PRBox as well as included antimycobacterial-positive controls and, importantly, to identify new scaffolds that could lead to new drugs against *M. ulcerans* viability (e.g., MMV1581558 and possibly also fusidic acid and GW611920X) and/or mycolactone production (e.g., pradigastat).

## Supplementary Material

Reviewer comments

## SUPPLEMENTAL MATERIAL

Fig. S1 spectrum.03968-25-s0001.pdf Results for reclassified compounds.

Table S1 spectrum.03968-25-s0002.xlsx Preliminary testing: effect of 400 MMV PRBox compounds on mycolactone expression.

OPEN PEER REVIEW reviewer-comments.pdf An accounting of the reviewer comments and feedback.

ASM does not own the copyrights to Supplemental Material that may be linked to, or accessed through, an article. The authors have granted ASM a non-exclusive, world-wide license to publish the Supplemental Material files. Please contact the corresponding author directly for reuse.
