# Supplementary material for: Activity of MMV Pandemic Response Box compounds against Mycobacterium ulcerans viability and mycolactone production
Source: Microbiol Spectr. 2026 Aug 19;14(10):e03968-25. doi: 10.1128/spectrum.03968-25 (PMC13689480; doi:10.1128/spectrum.03968-25)
Supplement: Supplementary file 1 [file reviewer-comments.pdf]

ASM | Microbiology Spectrum

Activity of MMV Pandemic Response Box compounds against Mycobacterium ulcerans viability and mycolactone production

Louisa Warryn and Daniel Paris

Corresponding Author(s): Louisa Warryn, Schweizerisches Tropen- und Public Health-Institut

Review Timeline:

Submission Date:	December 9, 2025
Editorial Decision:	April 3, 2026
Revision Received:	June 1, 2026
Editorial Decision:	June 22, 2026
Revision Received:	July 17, 2026
Accepted:	July 20, 2026

Editor: Silvia Cardona

Reviewer(s): The reviewers have opted to remain anonymous.

Transaction Report:

(Note: With the exception of the correction of typographical or spelling errors that could be a source of ambiguity, letters and reports are not edited. The original formatting of letters and referee reports may not be reflected in this compilation.)

DOI: https://doi.org/10.1128/spectrum.03968-25

Re: Spectrum03968-25 (Activity of MMV Pandemic Response Box compounds against Mycobacterium ulcerans viability and mycolactone production)

Dear Dr. Louisa Warryn:

Thank you for submitting your manuscript to Microbiology Spectrum. Your article has been reviewed by two experts in the field. Both reviewers found the findings interesting and have provided comments that, if addressed, will improve the quality of the work substantially.

Their recommendations are provided below.

Please return the manuscript within 60 days; if you cannot complete the modification within this time period, please contact me. If you do not wish to modify the manuscript and prefer to submit it to another journal, notify me immediately so that the manuscript may be formally withdrawn from consideration by Spectrum.

Revision Guidelines
To submit your modified manuscript, log into the submission site at https://spectrum.msubmit.net/cgi-bin/main.plex. Go to Author Tasks and click the appropriate manuscript title to begin. The information you entered when you first submitted the paper will be displayed; update this as necessary. Note the following requirements:

• Upload point-by-point responses to the issues raised by the reviewers in a file named "Response to Reviewers," NOT in your cover letter.
• Upload a compare copy of the manuscript (without figures) as a "Marked-Up Manuscript" file.
• Upload a clean .DOC/.DOCX version of the revised manuscript and remove the previous version.
• Each figure must be uploaded as a separate, editable, high-resolution file (TIFF or EPS preferred), and any multipanel figures must be assembled into one file.
• Any supplemental material intended for posting by ASM should be uploaded with their legends separate from the main manuscript. You can combine all supplemental material into one file (preferred) or split it into a maximum of 10 files with all associated legends included.

For complete guidelines on revision requirements, see our Submission and Review Process webpage. Submission of a paper that does not conform to guidelines may delay acceptance of your manuscript.

Data availability: ASM policy requires that data be available to the public upon online posting of the article, so please verify all links to sequence records, if present, and make sure that each number retrieves the full record of the data. If a new accession number is not linked or a link is broken, provide Spectrum production staff with the correct URL for the record. If the accession numbers for new data are not publicly accessible before the expected online posting of the article, publication may be delayed; please contact production staff (Spectrum@asmusa.org) immediately with the expected release date.

Publication Fees: For information on publication fees and which article types are subject to charges, visit our website. If your manuscript is accepted for publication and any fees apply, you will be contacted separately about payment during the production process; please follow the instructions in that e-mail. Arrangements for payment must be made before your article is published.

ASM Membership: Corresponding authors may join or renew ASM membership to obtain discounts on publication fees. Need to upgrade your membership level? Please contact Customer Service at Service@asmusa.org.

The ASM Journals program strives for constant improvement in our submission and publication process. Please tell us how we can improve your experience by taking this quick Author Survey.

Thank you for submitting your paper to Spectrum.

Sincerely,
Silvia Cardona
Editor
Microbiology Spectrum

Reviewer #1 (Comments for the Author):

Line 71: It seems that there is a typographical error and the word should be "engineer" and not "engender".

Line 185: The authors mentioned time kill assays which used the resazurin-based system, which was also used for the initial screening. Although they do refer to an earlier study where CFU counts and the metabolic readout correlated, in this case the assay is looking at the effects of many different molecules from distinct chemical classes. Therefore, the inclusion of CFU counts at the end of exposure period can provide substantial support for the results.

Figure 6 and associated text: It is clear that there are changes in mycolactone accumulation profiles as compared to the metabolic status in M. ulcerans when the specific compounds were used in dose responsive testing. One question, was there any indication and changes in growth? Resazurin is a readout of metabolic activity which can sometimes vary based on drug exposure as compared to actual growth levels, whereas mycolactone would be more associated with cell density. Therefore, it would be nice to see a clear correlation to show that there was no effect in cell number when mycolactone synthesis or levels were decreased on compound exposure.

Line 439: Again, it would be nice to see some correlation of CFU counts (or other growth assay) as compared to mycolactone levels on indole-2-carboxamide GW611920X (the main lead compound identified in the screen). This would provide a thorough validation of the assays and the results.

Reviewer #3 (Comments for the Author):

Introduction. It would be good to indicate global BU prevalence and current drugs/regimens used for BU and their shortcomings.

Line 114. Are M. ulcerans MICs typically expressed as concentrations effecting a 50% reduction in signal rather than 90%?

Line 117. Was mycolactone assessment also performed in duplicate?

Line 306. Is sequestration of mycolactone assumed to be the mostly likely MOA for compounds showing a reduction in the toxin? This is worthy of a brief discussion along with the probability of identifying drug-like PKS inhibitors. Are there any PKS inhibitors that can serve as positive controls?

Table 1 and 2. I would suggest using % inhibition instead of % activity (and use negative values for those that are below zero.)

Line 428. Is the screening platform significantly different from that in reference #9? Validation would include MICs below achievable exposures of current clinical drugs used for BU - rifampin, steptomycin and clarithromycin. Were these included as positive controls for growth inhibition? If so, their MICs should be stated.

Conclusions.

It might be noted that all classes of protein synthesis inhibitors demonstrated some degree of mycolactone suppression: macrolides, aminoglycosides, tetracyclines and fusidic acid.

Response to reviewers.

Reviewer #1 (Comments for the Author):

Line 71: It seems that there is a typographical error and the word should be "engineer" and not "engender".

We thank the reviewer for the observation. Although "engender" is correct in this context, for clarity we have reworded this sentence as follows: "*In this study, we assessed the potential of the PRBox collection as a source of active compounds for new BU drug development*."

Line 185: The authors mentioned time kill assays which used the resazurin-based system, which was also used for the initial screening. Although they do refer to an earlier study where CFU counts and the metabolic readout correlated, in this case the assay is looking at the effects of many different molecules from distinct chemical classes. Therefore, the inclusion of CFU counts at the end of exposure period can provide substantial support for the results.

We thank the reviewer for their comments, and we do agree that CFU counts can substantially corroborate our results. For the most part, the rather slow growth rate of *M. ulcerans* was the main reason we opted for the resazurin time-kill assay, as it could give comparable results within a month as opposed to the 3 – 6 months that CFU enumeration would have required. In this case, we used the resazurin assay to help narrow things down from 16 to a more manageable number. However, the best 5 included two IPA compounds (which as we mentioned in the manuscript, is already a well-studied compound class, including by our group (Ref #14)), and sutezolid (which already had been tested by others both *in vitro* and *in vivo* (Ref #13)). We therefore did not think these merited further tests. We unfortunately did not have sufficient compound volumes to do additional tests here for alexidine and MMV1581558, after running the mycolactone assays (we only reserved a little where we could for any additional tests requested during peer review).

Figure 6 and associated text: It is clear that there are changes in mycolactone accumulation profiles as compared to the metabolic status in M. ulcerans when the specific compounds were used in dose responsive testing. One question, was there any indication and changes in growth? Resazurin is a readout of metabolic activity which can sometimes vary based on drug exposure as compared to actual growth levels, whereas mycolactone would be more associated with cell density. Therefore, it would be nice to see a clear correlation to show that there was no effect in cell number when mycolactone synthesis or levels were decreased on compound exposure.

Regarding other growth assays, we considered $OD_{600\ nm}$ measurements. However, *M. ulcerans* cultures display variable levels of aggregation that make such biomass-based measurements as a readout for drug screening not very precise. Although there are possibilities of dispersing these aggregates (e.g. through bead-beating), the *M. ulcerans* ECM is disrupted by such

treatments, making the bacteria more susceptible to drugs than they typically would be. Nevertheless, we have now attempted to include $OD_{600\ nm}$ measurements alongside resazurin and mycolactone determination. Unfortunately, due to the limited volume of compounds available at the outset, we did not have sufficient volumes for all the compounds to do these additional tests with (we did attempt to get more but given the time needed to grow the bacteria to log phase and then do the 3-week assay, we elected to continue with what we had at hand). We have updated Fig. 6 to include these results, but we did not have sufficient volumes to retest the compounds shown in Fig. 4.

Line 439: Again, it would be nice to see some correlation of CFU counts (or other growth assay) as compared to mycolactone levels on indole-2-carboxamide GW611920X (the main lead compound identified in the screen). This would provide a thorough validation of the assays and the results.

As we mentioned above, we have now done OD_{600} measurements alongside resazurin reduction and mycolactone measurement. Interestingly, although all the other compounds showed $OD_{600\ nm}$ readings that mirrored the mycolactone and/or resazurin measurements in line with our previous conclusions (updated Fig. 6), for GW611920X, the reduction in mycolactone seen was indeed due to a reduction in biomass, even though the resazurin results suggested otherwise.

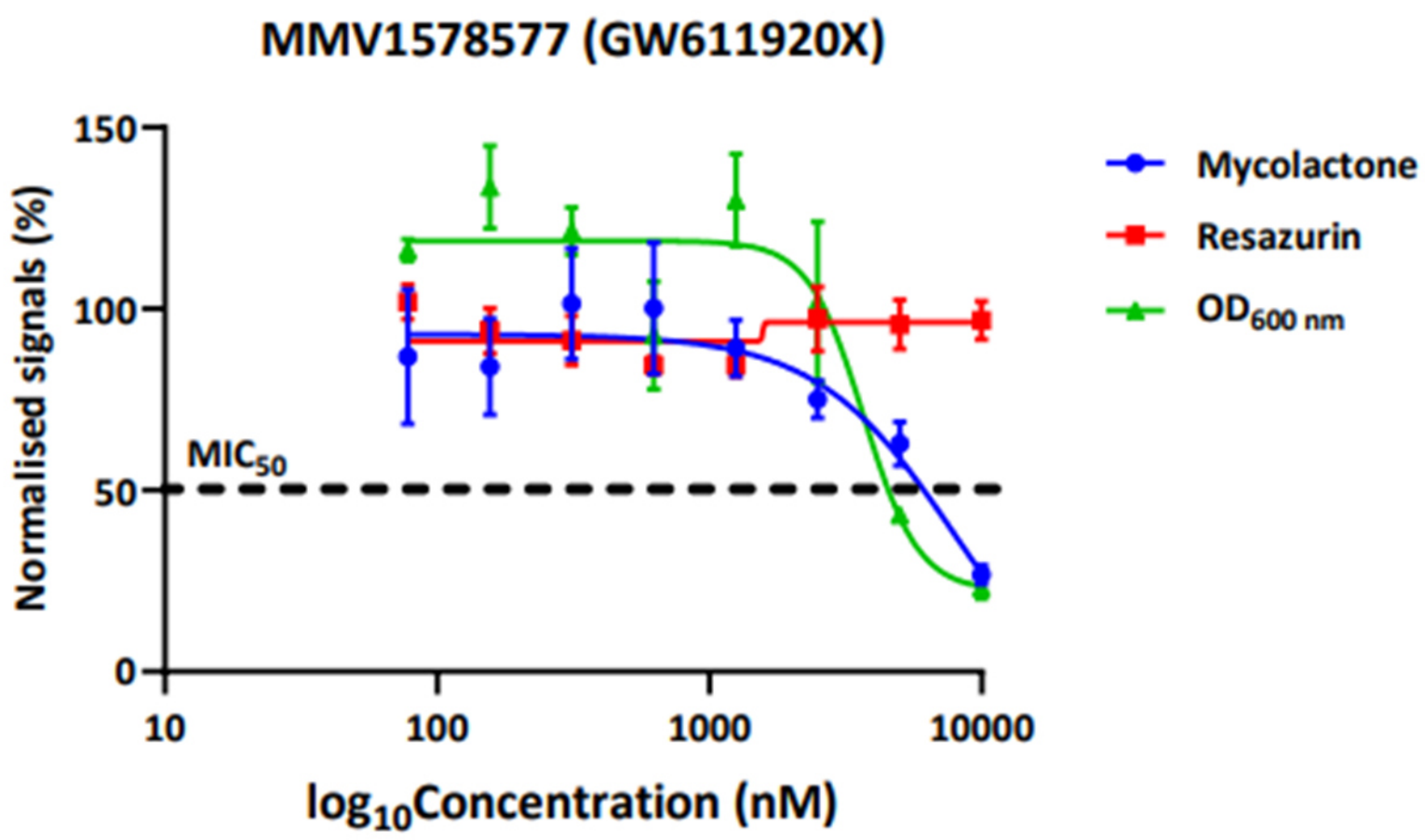

Reviewer #3 (Comments for the Author):

Introduction. It would be good to indicate global BU prevalence and current drugs/regimens used for BU and their shortcomings.

We thank the reviewer for this observation. We have now included these details in the introduction.

Line 114. Are M. ulcerans MICs typically expressed as concentrations effecting a 50% reduction in signal rather than 90%?

Reductions of 50%, 90%, and 99% have all been mentioned in the literature. In our experience, while 50% reduction can be directly read off the dose-response curve, 90% reduction often needs to be extrapolated from the curve (with GraphPad Prism, for instance), except for highly active compounds such as Q203 where IC_{90} or IC_{99} values can be directly read off the curve.

Line 117. Was mycolactone assessment also performed in duplicate?

Yes they were. We apologise for the omission. We have now included this in the text.

Line 306. Is sequestration of mycolactone assumed to be the mostly likely MOA for compounds showing a reduction in the toxin? This is worthy of a brief discussion along with the probability of identifying drug-like PKS inhibitors. Are there any PKS inhibitors that can serve as positive controls?

It is highly likely that different compounds that reduce mycolactone levels do so via different mechanisms, however at this time, we have not done any formal MOA determination for these compounds. We only surmised this mechanism for fenretinide based on some published literature. Given its high lipophilicity and reported propensity to accumulate in fatty tissue, we thought fenretinide might have some affinity for lipids, and with mycolactone being a lipid-like molecule, we thought that an association between fenretinide and mycolactone might be possible.

Regarding PKS inhibitors, this is something we have been considering, since mycolactone is synthesised by PKSs. Indeed, we are following up with this line of inquiry and have only just begun testing a small set of PKS inhibitors provided to us by a collaborator. It is entirely possible that a PKS inhibitor could suppress mycolactone production, but as we have not yet tested this hypothesis, we cannot comment on their suitability as positive controls.

Table 1 and 2. I would suggest using % inhibition instead of % activity (and use negative values for those that are below zero.)

We thank the reviewer for this suggestion. We have now changed Tables 1 and 2 accordingly.

Line 428. Is the screening platform significantly different from that in reference #9? Validation would include MICs below achievable exposures of current clinical drugs used for BU - rifampin, steptomycin and clarithromycin. Were these included as positive controls for growth inhibition? If so, their MICs should be stated.

No, the screening platform is essentially the same as described in reference #9. The only difference is that we found that incubating the plates with resazurin for 72 h at 30°C rather than 24 h at 37°C as stated in reference #9 gave identical results and was more suited to the established biosafety protocols in our BSL-3 facility.

We did include rifampicin as a positive control initially, but we decided that the inclusion of known antimycobacterials in the MMV PRBox would serve as suitable internal controls for our assessments. In those initial tests, we obtained MIC_{50} values of 0.15 – 0.97 µM for the three *M. ulcerans* strains (0.34 µM for S1013, 0.15 µM for S1252, and 0.97 µM for S1324), which is in line with reported *in vitro* MIC for rifampicin (0.122 – 0.984 µM). We have now included these results in the manuscript.

We retested rifampicin, clarithromycin, and streptomycin against strain S1013 and obtained MIC_{50} values of 0.31 µg/ml (rifampicin), 0.59 µg/ml (clarithromycin), and 0.52 µg/ml (streptomycin) which are in line with reported *in vitro* MIC for rifampicin (0.1 – 0.81 µg/ml), clarithromycin (0.125 – 1.25 µg/ml), and streptomycin (0.33 – 1.10 µg/ml) (Ref #24).

Conclusions.

It might be noted that all classes of protein synthesis inhibitors demonstrated some degree of mycolactone suppression: macrolides, aminoglycosides, tetracyclines and fusidic acid.

This is an interesting conclusion and we thank the reviewer for it. This was an exploratory study and in our ongoing follow-up study, we are assessing several members of these drug families (since we only had single representatives of these drug classes in the MMV PRBox), in addition to the PKS inhibitors, to see which scaffolds give the best mycolactone suppression. We feel that this additional assessment is necessary before we can make conclusions about drug classes that suppress mycolactone.

Re: Spectrum03968-25R1 (Activity of MMV Pandemic Response Box compounds against Mycobacterium ulcerans viability and mycolactone production)

Dear Dr. Louisa Warryn:

Please accept my apologies for the lengthy review process. While both reviewers found the revised version improved, one reviewer has an additional comment that must be addressed for final acceptance.

I also read the revised version and agree with the reviewer that the claim in the abstract, "reduced mycolactone expression without affecting bacterial viability," requires additional experimental evidence. The data in Figure 6 indeed suggest that reduced mycolactone is due to reduced biomass rather than to specific activity against mycolactone synthesis. In addition, the observation of reduced growth with no effect on bacterial metabolism needs to be revised. Spectrum's editorial policies are based on technical quality that supports the experimental evidence, even if the results are negative or less exciting.

The specific comments by the reviewer are found below.

Please return the manuscript within 60 days; if you cannot complete the modification within this time period, please contact me. If you do not wish to modify the manuscript and prefer to submit it to another journal, notify me immediately so that the manuscript may be formally withdrawn from consideration by Spectrum.

Revision Guidelines
To submit your modified manuscript, log into the submission site at https://spectrum.msubmit.net/cgi-bin/main.plex. Go to Author Tasks and click the appropriate manuscript title to begin. The information you entered when you first submitted the paper will be displayed; update this as necessary. Note the following requirements:

• Upload point-by-point responses to the issues raised by the reviewers in a file named "Response to Reviewers," NOT in your cover letter.
• Upload a compare copy of the manuscript (without figures) as a "Marked-Up Manuscript" file.
• Upload a clean .DOC/.DOCX version of the revised manuscript and remove the previous version.
• Each figure must be uploaded as a separate, editable, high-resolution file (TIFF or EPS preferred), and any multipanel figures must be assembled into one file.
• Any supplemental material intended for posting by ASM should be uploaded with their legends separate from the main manuscript. You can combine all supplemental material into one file (preferred) or split it into a maximum of 10 files with all associated legends included.

For complete guidelines on revision requirements, see our Submission and Review Process webpage. Submission of a paper that does not conform to guidelines may delay acceptance of your manuscript.

Data availability: ASM policy requires that data be available to the public upon online posting of the article, so please verify all links to sequence records, if present, and make sure that each number retrieves the full record of the data. If a new accession number is not linked or a link is broken, provide Spectrum production staff with the correct URL for the record. If the accession numbers for new data are not publicly accessible before the expected online posting of the article, publication may be delayed; please contact production staff (Spectrum@asmusa.org) immediately with the expected release date.

Publication Fees: For information on publication fees and which article types are subject to charges, visit our website. If your manuscript is accepted for publication and any fees apply, you will be contacted separately about payment during the production process; please follow the instructions in that e-mail. Arrangements for payment must be made before your article is published.

ASM Membership: Corresponding authors may join or renew ASM membership to obtain discounts on publication fees. Need to upgrade your membership level? Please contact Customer Service at Service@asmusa.org.

The ASM Journals program strives for constant improvement in our submission and publication process. Please tell us how we can improve your experience by taking this quick Author Survey.

Thank you for submitting your paper to Spectrum.

Sincerely,
Silvia Cardona
Editor
Microbiology Spectrum

Reviewer #1 (Comments for the Author):

I thank the authors for providing detailed responses to the reviewers' comments. However, one important question remains. In almost all cases where mycolactone production was reduced, bacterial growth was also inhibited. Do the authors have any evidence indicating whether these effects can be uncoupled? One of the stated goals of the study was to identify compounds that specifically inhibit mycolactone production without affecting bacterial viability (abstract). If growth is substantially reduced (and maybe viability), a concomitant decrease in mycolactone production would be expected, making it difficult to determine whether the compounds directly target mycolactone biosynthesis or simply reduce toxin production as a consequence of impaired growth.

As the authors note, the case of GW611920X is particularly interesting. Both growth and mycolactone production appear to be reduced, whereas the resazurin assay did not indicate a reduction in viability. This discrepancy raises questions regarding the interpretation of the resazurin data. If growth is indeed impaired while reducing activity is maintained, this could reflect compensatory metabolic activity or potential interference with the assay itself. It is unclear whether the compounds were tested for direct interactions with the resazurin assay in the absence of cells.

I believe these points require further clarification. While the data convincingly demonstrate that the mycolactone capture assay is effective for screening for recued production levels due to compounds, the evidence supporting the identification of compounds that specifically inhibit mycolactone production, independent of effects on bacterial growth, does not yet appear fully convincing.

Response to reviewers.

Reviewer #1 (Comments for the Author):

I thank the authors for providing detailed responses to the reviewers' comments. However, one important question remains. In almost all cases where mycolactone production was reduced, bacterial growth was also inhibited. Do the authors have any evidence indicating whether these effects can be uncoupled? One of the stated goals of the study was to identify compounds that specifically inhibit mycolactone production without affecting bacterial viability (abstract). If growth is substantially reduced (and maybe viability), a concomitant decrease in mycolactone production would be expected, making it difficult to determine whether the compounds directly target mycolactone biosynthesis or simply reduce toxin production as a consequence of impaired growth.

We acknowledge that the effect of test compounds on mycolactone biosynthesis independent of bacterial growth/viability might be difficult to determine, and this was why we made the distinction between Group 3 and 4 compounds, and further subdivided Group 3 compounds into those which only had early effects on resazurin and those which had only late effects. We hypothesised that any of these could be expected to affect mycolactone production. We then focused on the Group 2 compounds which did not show reduction in growth (either by resazurin and subsequently by OD as suggested by the reviewer) but did show a reduction in detected mycolactone as being potentially affecting mycolactone production. A follow-up study would, of course, need to be done to ascertain whether or not mycolactone production is impaired and the mechanisms that cause such impairment, but this is beyond the scope of the current study.

For subsequent studies, we are incorporating OD measurements as well into the screening pipeline, since it can be readily done in similar throughput as the other assays. We again thank the reviewer for suggesting this.

As the authors note, the case of GW611920X is particularly interesting. Both growth and mycolactone production appear to be reduced, whereas the resazurin assay did not indicate a reduction in viability. This discrepancy raises questions regarding the interpretation of the resazurin data. If growth is indeed impaired while reducing activity is maintained, this could reflect compensatory metabolic activity or potential interference with the assay itself. It is unclear whether the compounds were tested for direct interactions with the resazurin assay in the absence of cells.

Regarding GW611920X, we looked critically through all our available data again and agreed that GW611920X and eravacycline must be reclassified as Group 3 compounds. We have therefore modified Figure 6 to show only those compounds where only mycolactone levels but not OD or resazurin were reduced. GW611920X and eravacycline are in the new supplementary figure S1. We have modified the text to explain these observations.

Regarding the reviewer's question, we also checked to see if GW611920X could reduce resazurin in the absence of cells, but this was not the case, as shown in the figure below. At this juncture, it is unclear why OD and resazurin gave discordant results for this compound.

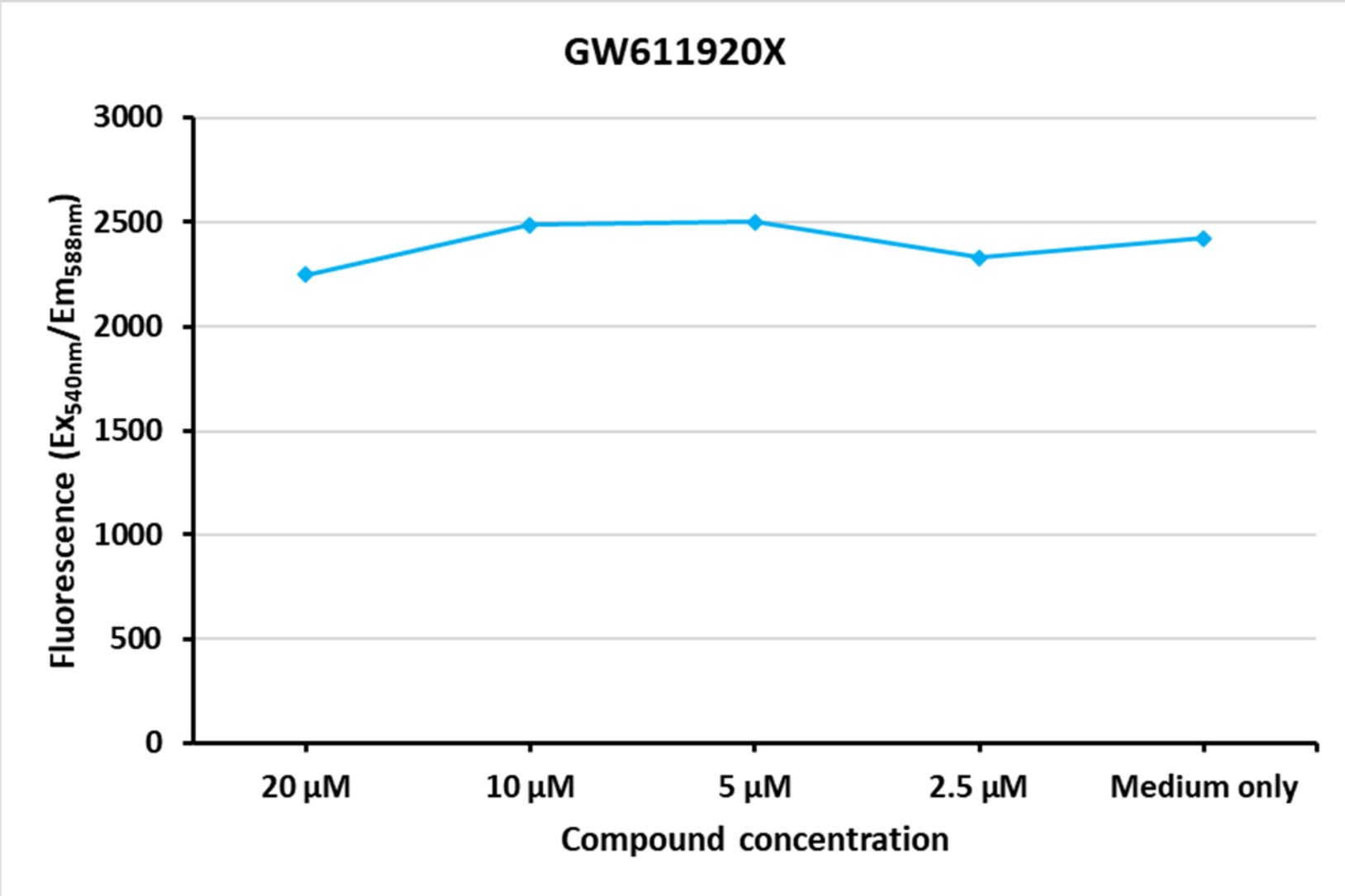

In all this, we realised that we were a bit too focused on resolving the conundrum that GW611920X presented that we overlooked pradigastat, so we have now rectified this oversight as well.

I believe these points require further clarification. While the data convincingly demonstrate that the mycolactone capture assay is effective for screening for recued production levels due to compounds, the evidence supporting the identification of compounds that specifically inhibit mycolactone production, independent of effects on bacterial growth, does not yet appear fully convincing.

We thank the reviewer for their observations, and for supporting the utility of our screening platform, as well as for taking the time to critically review our work. We hope that our additional modifications would prove to be more convincing.

Re: Spectrum03968-25R2 (Activity of MMV Pandemic Response Box compounds against Mycobacterium ulcerans viability and mycolactone production)

Dear Dr. Louisa Warryn:

Congratulations! Your manuscript has been accepted, and I am forwarding it to the ASM production staff for publication. Your paper will first be checked to make sure all elements meet the technical requirements. ASM staff will contact you if anything needs to be revised before copyediting and production can begin. Otherwise, you will be notified when your proofs are ready to be viewed.

Data Availability: ASM policy requires that data be available to the public upon online posting of the article, so please verify all links to sequence records, if present, and make sure that each number retrieves the full record of the data. If a new accession number is not linked or a link is broken, provide production staff with the correct URL for the record. If the accession numbers for new data are not publicly accessible before the expected online posting of the article, publication may be delayed; please contact ASM production staff immediately with the expected release date.

Publication Fees: For information on publication fees and which article types have charges, please visit our website. We have partnered with Copyright Clearance Center (CCC) to collect author charges. If fees apply to your paper, you will receive a message from no-reply@copyright.com with further instructions. For questions related to paying charges through RightsLink, please contact CCC at ASM_Support@copyright.com or toll free at +1-877-622-5543. CCC makes every attempt to respond to all emails within 24 hours.

ASM Membership: Corresponding authors may join or renew ASM membership to obtain discounts on publication fees. Need to upgrade your membership level? Please contact Customer Service at Service@asmusa.org.

Embargo Policy: A press release may be issued as soon as the manuscript is posted on the Spectrum Latest Articles webpage. The corresponding author will receive an email with the subject line "ASM Journals Author Services Notification" when the article is available online.

Cover Image Submissions: If you would like to submit a potential Cover Image, please email a file and a short legend to spectrum@asmusa.org. Please note that we can only consider images that (i) the authors created or own and (ii) have not been previously published. By submitting, you agree that the image can be used under the same terms as the published article. Image File requirements: TIF/EPS, 7.5 inches wide by 8.25 inches tall (at least 2,250 pixels wide by 2,475 pixels tall), minimum 300 dpi resolution (600 dpi preferred), RGB, and no figure elements, e.g., arrows or panel labels. The legend should be a short description of the image, 1-2 sentences recommended. Please download and use this interactive template in Adobe the https://journals.asm.org/pb-assets/html/asm-cover-sizing-tool.html to ensure that your proposed cover image meets our size requirements (https://journals.asm.org/pb-assets/pdf-text-excel-files/ASM-Interactive-Sizing-Cover-Template-1715689791.pdf).

The ASM Journals program strives for constant improvement in our submission and publication process. Please tell us how we can improve your experience by taking this quick Author Survey.

Thank you for submitting your paper to Spectrum.

Sincerely,
Silvia Cardona
Editor
Microbiology Spectrum
