# Supplementary material for: Activity of MMV Pandemic Response Box compounds against Mycobacterium ulcerans viability and mycolactone production
Source: Microbiol Spectr. 2026 Aug 19;14(10):e03968-25. doi: 10.1128/spectrum.03968-25 (PMC13689480; doi:10.1128/spectrum.03968-25)
Supplement: Supplementary file 2 [file spectrum.03968-25-s0001.pdf]

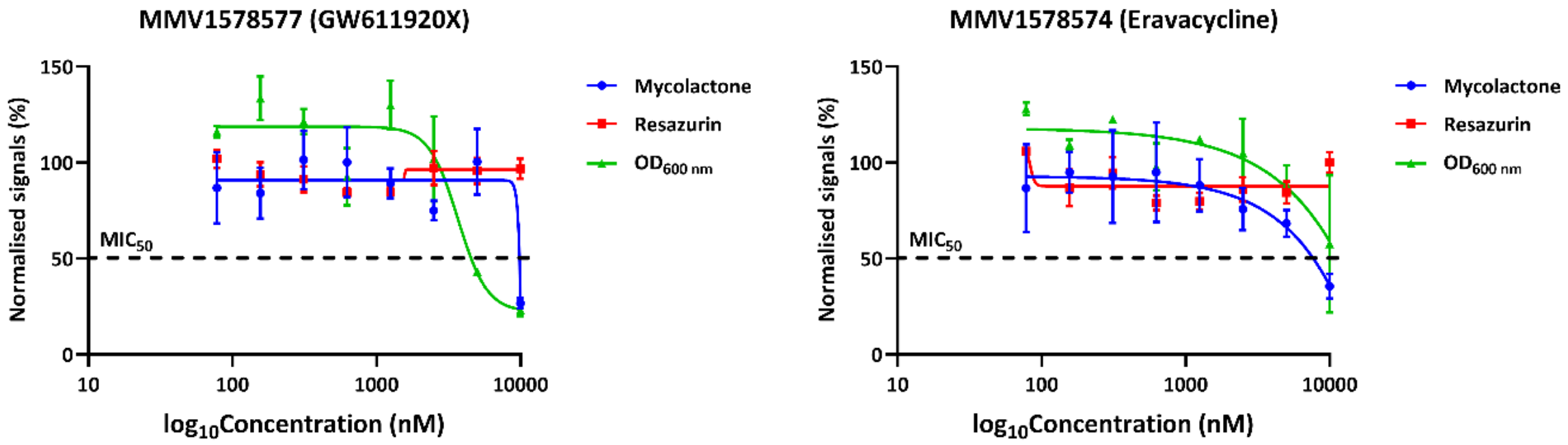

Figure S1. PRBox compounds Eravacycline and GW611920X. Dose ranges of test compounds were assessed for their effects on *M. ulcerans* viability (as determined by resazurin reduction and $OD_{600\ nm}$) and mycolactone detection (as determined by capture ELISA (10)). Graphs show means of triplicate experiments with error bars representing the SEM.
